# Advanced Red Meat Cooking Technologies and Their Effect on Engineering and Quality Properties: A Review

**DOI:** 10.3390/foods12132564

**Published:** 2023-06-30

**Authors:** Bandar M. Alfaifi, Saleh Al-Ghamdi, Moath B. Othman, Ali I. Hobani, Gamaleldin M. Suliman

**Affiliations:** 1 Department of Agricultural Engineering, College of Food and Agriculture Sciences, King Saud University, P.O. Box 2460, Riyadh 11451, Saudi Arabia; balfaifi@ksu.edu.sa (B.M.A.); moath204@hotmail.com (M.B.O.); hobani@ksu.edu.sa (A.I.H.); 2 Department of Agricultural Engineering, Faculty of Agriculture, Foods & Environment, Sana’a University, Sana’a 13020, Yemen; 3 Department of Animal Production, College of Food and Agriculture Sciences, King Saud University, P.O. Box 2460, Riyadh 11451, Saudi Arabia; gsuliman@ksu.edu.sa

**Keywords:** red meat, cooking technologies, engineering properties, packaging, mathematical modeling

## Abstract

The aim of this review is to investigate the basic principles of red meat cooking technologies, including traditional and modern methods, and their effects on the physical, thermal, mechanical, sensory, and microbial characteristics of red meat. Cooking methods were categorized into two categories: traditional (cooking in the oven and frying) and modern (ohmic, sous vide, and microwave cooking). When red meat is subjected to high temperatures during food manufacturing, it undergoes changes in its engineering and quality attributes. The quality standards of meat products are associated with several attributes that are determined by food technologists and consumers based on their preferences. Cooking improves the palatability of meat in terms of tenderness, flavor, and juiciness, in addition to eliminating pathogenic microorganisms. The process of meat packaging is one of the important processes that extend the life span of meat and increase its shelf life due to non-exposure to oxygen during cooking and ease of handling without being exposed to microbial contamination. This review highlights the significance of meat cooking mathematical modeling in understanding heat and mass transfer phenomena, reducing costs, and maintaining meat quality. The critical overview considers various production aspects/quality and proposed methods, such as, but not limited to, hurdle technology, for the mass production of meat.

## 1. Introduction

Red meat refers to raw meat such as beef, lamb, pork, horsemeat, camel meat, and venison and may also include meat from other types of mammals. It is commonly red in color when it is raw due to its myoglobin content (a muscle cell pigment). Red meat is a rich source of proteins, fatty acids, iron, zinc, B vitamins, and many other important nutrients [1].

Recent advances in meat cooking and preservation technologies have played an essential role in increasing the production of red meat globally. This increase is anticipated to continue, especially in developing countries like China, India, Brazil, and Russia, due to increases in populations and incomes [2]. The total global meat production is about 337 million tons/annually, of which 206 million tons are red meat and 131 million tons are white meat, while the production of processed meat is 27 million tons [3].

Meats are subjected to different thermal processes before they reach consumers. Meat cooking is among such processes, as it changes the original color, texture, and palatability. Temperature and cooking time are the most important factors that lead to changes in the physical and structural properties of meat, which results in a change in nutritional value due to loss of nutrients [4].

Red meat is made of muscle fibers, known as myofibrils, that are linked together by connective tissue, such as collagen and elastin. Knowing the type and amount of connective tissue in meat is important in selecting the appropriate cooking method [1]. There are several methods of cooking red meats, including traditional ones, such as cooking in the oven and frying, and modern ones, such as sous vide cooking, microwaving, and ohmic cooking. Recently, interest has increased in using modern cooking techniques to reduce the costs of energy consumption, raise the production rate, and improve meat quality, as well as to change consumer behavior from preferring traditional cooking to ordering ready-to-eat meals [5]. During cooking, both muscle fibers and connective tissues shrink, destroying cell membranes. Moreover, proteins play a major role in changing the characteristics of cooked meat, which affects the tenderness and general acceptability of cooked meat [6,7,8]

Quality standards of meat products are associated with several physical, thermal, mechanical, and sensory properties that are usually determined by producers and consumers. Some of these properties are determined by consumer preferences for a specific food product and not others, and those properties are color, taste, smell, consistency, and texture. Hence, sensory properties are important in identifying the acceptability and palatability of cooked meat, as these properties can determine the quality of meat. On the other hand, the importance of thermal properties lies in determining and understanding the temperature distribution inside the meat during heat treatment [9]. Determination of these properties is important since they establish engineering elements for equipment design, manufacturing, processing, and control processes [10]. The meat cooking process aims to improve meat quality properties and reduce the development of microorganisms responsible for meat spoilage since meat is considered a quickly perishable food material due to its high moisture content and the presence of several nutritional elements that make it an appropriate environment for the development of microorganisms like bacteria, fungi, and yeasts. The process of meat packaging before and after cooking is regarded as one of the modern techniques that have been increasingly used, as it is used to preserve structural quality properties of meat in addition to conserving meat flavor, decreasing microbial risks, and reducing labor costs [11].

During meat cooking, heat and mass transfer occur because of the difference in temperature and moisture content between the product and the surrounding air, and moisture evaporates from the product’s surface [12]. There are many factors affecting heat and mass transfer, including cooking temperature, air flow velocity, type of cooked product, and cooking medium. Mathematical modeling of the meat cooking process helps in studying the effect of these factors to understand the processes of mass and heat transfer, which may help in the development and design of the equipment used and planning the manufacturing process. This would thus improve the quality and flavor of cooked meat, extend its shelf life, reduce cooking losses, and control variables during heat treatment [13].

This study aims to review the basic principles of red meat cooking techniques, including traditional and modern cooking methods and their impact on the physical, mechanical, thermal, sensory, and microbial properties of meat as well as modern techniques used to preserve cooked meat and model the cooking process.

## 2. Meat Cooking Methods

Cooking is defined as the science and art of preparing fresh food to be consumable after heat treatment over a specific time. The cooking process was discovered by chance due to a forest fire, at which point ancient humans noticed that grilled meat tasted better than raw meat. The method of cooking meat by grilling lasted for a long time, and over the passage of time, humans began to use plant leaves in the cooking process, followed by numerous developments in the cooking process, including the use of clay pots, ovens, and boiling water [14]. This was followed by rapid developments in food processing techniques and processes aimed at meeting the needs and desires of consumers, preserving meat quality for as long as possible, reducing labor costs, and saving energy consumption. These newer techniques included ohmic, microwave, and sous vide cooking [15].

The cooking method and conditions such as heating rate, cooking time, and temperature are among the most important factors leading to changes in the composition of meat, which results in a change in nutritional value due to a loss of nutrients [16]. The choice of cooking method depends on many factors, the most important of which are the cut of meat being cooked, the number of connective tissues, the size and shape of the meat, and consumer preference [17,18]

Compositional quality standards for meat products are associated with several physical, mechanical, and sensory properties. These properties are usually determined by food science and technology scientists, factories, and consumers. The properties are color, taste, smell, consistency, and texture, which can be perceived sensuously. The quality properties of cooked meat are also affected by many factors, the most important of which are the method of cooking, the method of heat transfer from the source to the piece of meat, and the cut of meat used in cooking [5]. Table 1 shows some red meat cooking methods at various cooking conditions in different published studies.

### 2.1. Traditional Cooking Methods

#### 2.1.1. Electric Oven Cooking

**Figure 1:**
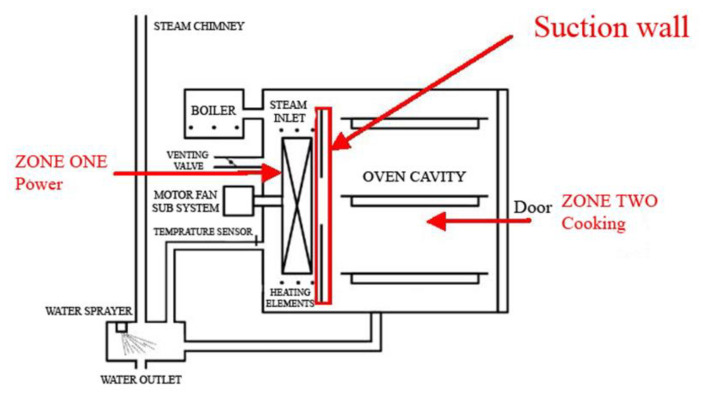
Electric oven layout adapted with modification from Burlon et al. [27] (Permission is obtained).

**Figure 2:**
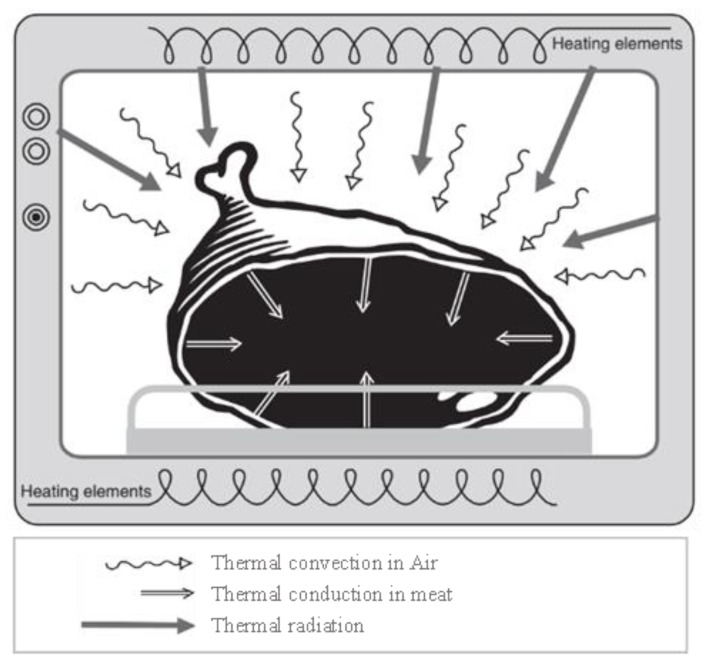
Illustration of heat transfer during meat cooking in an electric oven [17] (Permission is obtained).

The electric oven cooking method belongs to the traditional ones used to cook meat, where the temperature inside the oven reaches as high as 250 °C. During meat cooking in an electric oven, electrical energy is converted into thermal energy. Figure 1 shows a diagram of the electric oven, which consists of a heat source, a fan, switches, and sensor to control the temperature and time, and a rack to place the meat on; the heat source consists of a rod located on the upper and lower sides of the oven. In this type of cooking, heat is transferred to the meat through radiation, convection, and conduction [26]. Figure 2 shows an illustration of heat transfer during meat cooking in an electric oven by a combination of radiation, convection, and conduction [17].

There are many factors that affect cooking using the electric oven method, the most important of which are temperature, cooking time, components of the food, and the percentage of steam in the oven [5]. During oven cooking, the meat is dried from the outer surface to reduce the cooking time through natural and forced convection heating [28]. There are several studies that have discussed the effect of cooking red meat in an electric oven [19,28,29]. In his study of the effect of cooking with an electric oven on the sensory and physical properties of camel meat, Dawood [29] stated that the higher the animal’s age is, the longer the necessary cooking time, that the palatability of the meat of younger camels is better than the palatability of the meat of older camels, and that juiciness decreases with the increase in the camel’s age. In the study of the effect of aging and oven cooking on the protein consistency and texture of beef muscles at 70–80 °C, Palka [28] noticed the occurrence of deterioration and damage in proteins, and at temperatures above 80 °C, damage occurred in most proteins. Protein damage is considered one of the most important factors that increase protein hardness and thus affects the tenderness of meat due to its effect on fiber hardness. Therefore, electric oven cooking can be combined with steam for more tender meat, where the moisture from steam can prevent meats’ outer surfaces from drying out and shrinking, maintaining the tenderness and juiciness of the cooked meat [30]. On the other hand, Hobani et al. [19] reported that pH, cooking loss, density, and color components of camel meat were significantly affected by the electric oven cooking method. The authors added that cooking by the electric method had prompted a loss of water, minerals, as well as free amino acids of protein fibers due to low thermal conductivity resulting from the dry, hot air used for cooking.

#### 2.1.2. Frying

Frying is a traditional cooking technique where cooking oil is used as a medium for heat transfer as it is in direct contact with the meat. During fry cooking, heat transfer also occurs between the frying pan and the meat through direct contact between them [5]. There are many factors affecting the efficiency of the fry cooking method, the most important of which are the components of the food material, the surface area of meat exposed to oil, and the initial oil temperature [31].

During fry cooking, a piece of meat undergoes four basic stages. In the first stage, a heat transfer occurs through convection between the meat’s surface and the surrounding ambient heat, while in the second stage, water begins to leave the meat through evaporation, in addition to other changes caused by thermal convection between the oil and meat, and natural convection is transformed to forced convection. In the third stage, the temperature begins to rise, and a hard layer forms on the surface of the meat. Finally, in the fourth stage, the temperature increases in the center of the piece of meat until it is well-cooked [31].

Many changes occur during the cooking of meat in hot oil, such as a rapid loss of moisture content, rapid protein denaturation, oil absorption, discoloration, and the formation of a hard-outer layer on the meat. This method is also characterized by a very short cooking time [32]. Sosa-Morales et al. [33] stated in their study on mass and heat transfer and the quality aspects of frying pork in cooking oil that the process of frying depends on the temperature of the frying oil, and the thermal diffusion remain constant throughout the cooking time, while the coefficient of thermal conductivity decreases with increased frying time due to the loss of moisture content in the meat.

### 2.2. Modern Cooking Methods

#### 2.2.1. Ohmic Cooking

The basic principle of ohmic heating is that electrical energy is converted into thermal energy within the food product [34]. When an electric current passes through a conductor, the charges stimulate the molecules, which leads to a rise in the temperature inside the metal conductor due to the movement of electrons, and in food materials, the charges are in the form of ions like proteins that move to the electrode and heat the food material [35,36]. It was noted that the higher the voltage difference, the higher the temperature will be, and thus there will be an increase in the rate of heat, as the heat produced inside the meat is distributed homogeneously and results in changes in the physical and mechanical properties of the food material [37]. There are many benefits of ohmic cooking, including short cooking time and a decrease in energy consumption [38]. Parrott [39] also stated that there are many advantages of ohmic heating, including that it does not require a surface for thermal transfer, making it a fast method, and the product remains thermally homogeneous. Ohmically processed food materials have a longer shelf life, preserved color and nutritional value, and are perceived as environmentally friendly technologies compared to conventional methods [37].

**Figure 3:**
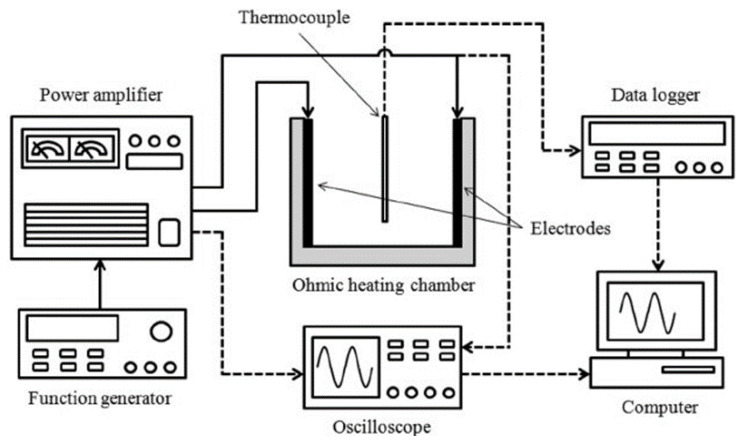
Schematic diagram of ohmic heating system reproduced from Lee et al. [41] (Permission is obtained).

Figure 3 shows an illustration of the ohmic cooking system, which consists of a cuboid container, stainless steel electrodes covered with a titanium coating, a power supply, devices to control voltage differences and current, a data logger, a thermocouple, and a transformer. In this method, the piece of meat is placed in a cooking container through which an alternating electric current with a certain voltage difference and current intensity is passed, which is controlled by the voltage controller, and then heat is generated in the meat [40].

There are several studies on the ohmic cooking of meat. Bozkurt and Icier [25] investigated the effect of ohmic cooking on the quality traits of beef. The meat was treated with an electric field intensity of 20, 30, and 40 V/c and fat contents of 2%, 9%, and 15%. The study found that ohmic cooking was significantly faster than traditional cooking. Moreover, the samples cooked by the ohmic method were more stable and cohesive than samples cooked using the traditional method. The cooking loss in ohmic cooking was 9.6%, while in traditional cooking, it was 31.39%. In another study by Aydin et al. [42], the effect of ohmic and traditional (water bath) cooking methods on the microbiological, sensory, and color values of fish Pâté was investigated. The findings showed that the cooking time for meat using ohmic cooking was less than the traditional method. Furthermore, the sensory properties of ohmic cooking were superior to those of the traditional method with respect to appearance, flavor, and odor scores. Ghnimi et al. [43] investigated the impact of ohmic cooking on the electrical conductivity of ground beef. The study found that the components of ground meat played a significant role in increasing the heating rate during ohmic cooking, resulting in an increase in the electrical conductivity of the meat and ultimately improving its quality attributes. Yildiz and Dere [44] also studied the effect of infrared cooking on meatballs that were pre-cooked with ohmic heating, and it was noted that ohmic heating cooks homogeneously but does not give a dark color on the surface of the meatballs, and the appearance of the meatballs was improved after infrared cooking. Therefore, ohmic cooking and infrared cooking were combined to maintain a better quality of the sensory properties of meatballs.

#### 2.2.2. Sous Vide Cooking

The Advisory Committee for Cooks defined sous vide cooking as food being placed in vacuumed and sealed plastic pouches and cooked in a water bath for longer than usual times at a precisely controlled temperature [45]. Meat is typically cooked at a low temperature of 65–90 °C for a time of 2–8 h, depending on the food ingredients [46]. It was originally a French method that spread globally due to its convenience and ease of application and can also be used to market cooked meat to restaurants. There are many advantages of this method, including reducing water loss and preserving volatile flavors and odors, thus preserving the sensory quality of meat [47]. Furthermore, Mortensen et al. [48] also indicated that the method of cooking in vacuumed plastic bags with a constant internal temperature in the meat maintains the color gradient and texture inside the piece of meat, and it also maintains the flavor and freshness and gives texture and homogeneity to the color of the meat.

Many changes occur in the physical, mechanical, and sensory properties of meat, and cooking meat products with sous vide yields better results than traditional methods due to the use of low temperatures for cooking versus the high temperatures traditional methods rely on. The denaturation of and damage to protein fibers, color changes, and sensory properties in the sous vide are lower compared to conventional methods [49].

**Figure 4:**
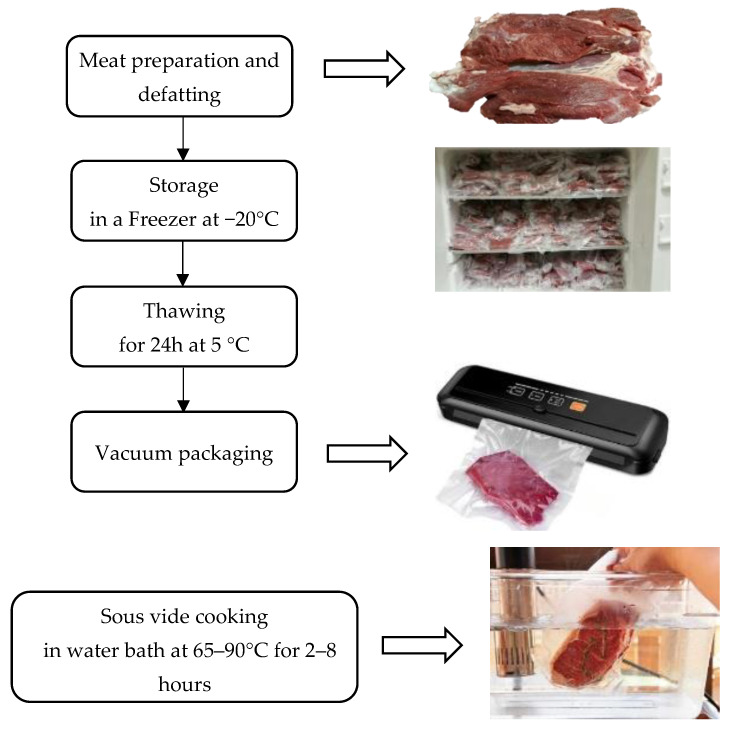
Schematic chart of Sous vide cooking.

It is recommended that when cooking beef and lamb using sous vide, the temperature should be between 58 and 63 °C for 10 to 48 h [47]. It has recently been noted that using vacuum packaging to cook meat at a low temperature over a long time has become one of the most used cooking methods. Figure 4 shows a diagram of the sous vide cooking method.

There are several studies on the cooking of red meat using the sous vide method. Becker et al. [50] cooked pork at low temperatures (53 °C, 58 °C, and 60 °C) for 20 h and studied its improvement in physicochemical and sensory properties. The results showed a significant effect on the tenderness of the meat. It has also been shown that when meat is cooked at 60 °C over time, the meat’s tenderness decreases and its juiciness increases and its quality is acceptable when cooked at 53 °C. Vaudagna et al. [51] cooked beef using the sous vide method to study the effect of low temperature and long times on quality properties and stable storage. They observed that the shear force and cooking losses decreased at temperatures between 50 and 65 °C at times of 90–360 min, and the temperature was sufficient to kill microbes such as Clostridium and polonium, and the microbial quality of the product remained acceptable.

#### 2.2.3. Microwave Cooking

The microwave cooking method is one of the methods that should be addressed in this review as an advanced cooking method. Microwave is a form of electromagnetic waves with frequencies ranging from 300 MHz to 300 GHz. Most industrial and commercial microwave ovens operate at frequencies of 915 MHz and 2450 MHz, which correspond to wavelengths of 32.8 cm and 12.2 cm, respectively. In contrast, domestic microwave ovens only operate at a frequency of 2450 MHz [52].

**Figure 5:**
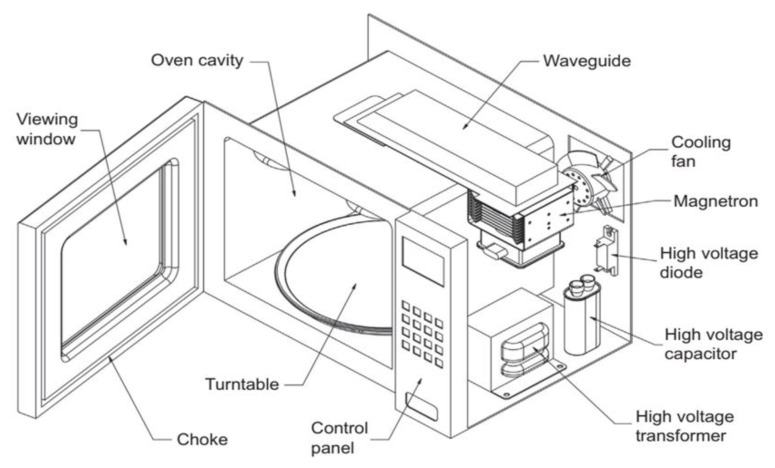
Schematic diagram of microwave heating system reproduced with permission from Cooper [53] (Permission is obtained).

Figure 5 shows the components of a domestic microwave oven that is used in the cooking process. It consists of a cavity surrounded by metal walls with a door in the front, a voltage transformer to convert the low input voltage (110–220 volts) into high output voltage (3000–4000 volts), a magnetron to generate the microwave and a waveguide to direct the microwave to the cavity. In addition, a cooling fan motor is installed to protect the magnetron from overheating, a turning disc to improve heat distribution in the food, and a control panel [53].

Unlike conventional heating, where heat is transferred from outer surfaces to the interiors gradually, microwave heating occurs by the conversion of electromagnetic energy into thermal energy volumetrically within the meat due to dipolar and ionic mechanisms. The importance of these mechanisms arises when an electromagnetic wave passes through the meat. Polar molecules, such as water molecules, rotate, causing molecular friction and collisions, and dissolved charged molecules (ions), such as salt molecules, oscillate back and forth, generating heat throughout the meat [52].

There are many factors affecting the microwave cooking process, such as the physical, thermal, and dielectric properties of meat and its components. One significant component in meat is the moisture content, as water is among the polar molecules, and salt, as salt is among the dissolved charged molecules (ions), where they, dipoles and ions, play an important role in generating heat within meat processed in microwave ovens. A major advantage of using microwave cooking as compared to conventional cooking methods is the rapid generation of heat within the material. This advantage may help to reduce the quality degradation of meat and energy consumption [54].

Several studies have been conducted to investigate the potential of microwave red meat cooking. Early studies were reported in the 1950s by Causey et al. [55,56,57]. The growing demand for both consumers and meat manufacturers to improve the quality and safety of meat, throughput, and energy efficiency raised the interest in continuing to study microwave meat cooking until today. In terms of quality, physical properties, protein denaturation, microstructure, and volatiles were investigated for microwave yak meat cooking. The findings indicated that cooking yak meat with a microwave resulted in better texture and volatile properties but greater cooking loss and decolorization compared to traditional boiling cooking [58]. In terms of health concerns, microwave cooking showed the lowest amount of total volatile N-nitrosamines and polycyclic aromatic hydrocarbons, which are two of the most hazardous compounds for human health, in beef cocktail smokies compared to pan-frying and grilling cooking [59]. In terms of energy saving, microwave cooking had lower energy consumption than traditional cooking for beef burgundy cooking due to the reduced cooking time, 56% compared to traditional cooking [60].

Some concerns with microwave meat cooking still exist, such as uneven heating, absence of browning and crust formation, and unpreferable textural changes. However, these concerns can be overcome by accompanying microwave cooking with another cooking method, such as convection, induction, and radiative heating [61].

## 3. Effects of Cooking Methods on Meat Engineering and Quality Properties

### 3.1. Physical Properties

During the cooking of meat, many changes occur in its physical properties [5]. Studying the effect of cooking on the physical properties of meat is important to determine the extent of consumer acceptance of meat. Physical properties are considered one of the most important properties affecting the palatability of meat among consumers, including pH, cooking losses, water activity, and color.

#### 3.1.1. pH

The pH of meat is one of the most important physical properties that affect cooked meat quality characteristics, as it affects meat muscles. There are many factors that affect the pH of meat, the most important of which are the animal’s treatment before slaughter, the glycogen storage percentage in the muscles, the glycogen consumption, and the accumulation of lactic acid in the pre-slaughtering stage, as these factors affect meat tenderness and juiciness [62]. The pH of red meat ranges from 5.5–6.6 [4,63]. It was also noted that the pH of the meat of young animals is high, while the meat of large animals has a relatively low pH due to low glycogen [63].

There are many studies that have studied the effect of different cooking methods on pH, including the study of Aprisal et al. [64] on the physicochemical properties of ohmic-cooked beef balls with the addition of different levels of salt (2, 3, and 4%), where meat was cooked at 140 volts, and the pH was measured after cooking. The results showed that the pH increases with increasing salt levels. Karakaya et al. [65] studied the differences in physical properties between lamb, beef, goat, and rabbit meats. They noted that the acidity in lamb and goat meats is higher than in beef and rabbit and that cooking losses in lamb are lower than in other meats. They also found that the meat cooking losses are 33.2% in lamb, 33.9% in goat, 35% in beef, and 33.8% in rabbit meat. Table 2 shows the pH values of some types of red meat cooked with different methods.

#### 3.1.2. Cooking Loss

Cooking loss is a crucial factor that affects the meat industry because it can change the shape and protein levels in meat, leading to reduced eating quality and financial returns from the cooking process [67,68]. Cooking loss is defined as a mixture of liquids and solids lost from meat during cooking and depends on many factors: cooking temperature, cooking duration, connective tissue components, and muscle fibers. Cooking losses can be calculated based on the weight of meat before and after heat treatments, where water comes out of the meat in the form of liquid or vapor. At a temperature above 70 °C, the cooking loss increases in thermal treatments [8].

Cooking loss can be calculated by weighing meat steak samples before cooking, cooking the samples, drying them using drying paper, and weighing the samples again after cooking. Table 3 shows the cooking losses of some types of red meat cooked using different methods. The following relationship can be used to calculate cooking loss [19,69,70]:

(1) $$\mathrm{Cooking}\;\mathrm{loss}\;\left(\%\right)=\frac{m_{1}-m_{2}}{m_{1}}\times 100$$

where *m_1_* is the raw meat sample (g), and *m_2_* is the cooked meat sample (g).

Several studies have investigated the effects of different cooking methods on cooking losses of meat. Lorenzo et al. [24] conducted a study to determine the effect of four different cooking methods (grilling, microwave, roasting, and frying) on the physicochemical properties of foal meat. The authors observed that microwave cooking resulted in higher losses compared to the other cooking methods. In a study by Fabre et al. [76], the effect of different cooking methods (electric oven, sous vide, and grilling) on cooking losses in various cuts of beef was investigated. The results revealed that the electric oven cooking method had a significant impact on cooking losses. Tian et al. [77] compared the ohmic and water bath cooking methods on the structural quality properties of pork treated with different levels of fat (0–40%). The results showed that the cooking losses in the water bath cooking method were higher than in the ohmic cooking. In another study, Hobani et al. [19] reported a significant increase in cooking loss of camel meat when cooked using the sous vide method compared to the electric oven.

#### 3.1.3. Color

The color of meat is an important physical property that can affect the perceived quality and appeal of the meat to the consumer. The color of meat can be influenced by several factors, including the animal’s breed, age, and diet, the type of muscle fibers, the concentration of myoglobin pigments in the muscles, the amount of fat, and the cooking method used [78]. Different cooking methods can have varying effects on the color of meat, depending on factors such as the cooking time, the temperature, and the presence of oxygen. For example, grilling and frying can result in a browned or charred exterior and a pink interior, while roasting in an oven can also produce a browned exterior and pink interior but with a more even color throughout the meat [79]. On the other hand, some cooking methods, such as sous vide, can enhance the retention of the natural color of meat by cooking it at low temperatures for longer periods of time [80]. In addition to the cooking method, the color of meat can also be affected by the presence of additives such as spices and marinades, as well as the packaging and storage conditions [78]. However, cooking methods play a significant role in the final color of the meat. By understanding the effects of different cooking methods on the color of meat, manufacturers can adapt appropriate cooking techniques to achieve desired results.

Several studies have investigated the effect of different cooking methods on the physical properties of meat, including color. García-Segovia et al. [4] studied the effect of cooking methods on the color and mechanical properties of beef pectoral muscles and observed that the color of beef steaks cooked in a vacuum package tended to be red. Yildiz and Dere [44] investigated the effect of infrared and ohmic cooking on meatballs and found that combining these methods resulted in improved appearance and sensory properties. Oz et al. [66] evaluated the effects of different cooking methods, including deep fat frying, hot plate, boiling, microwave, panfrying with oil, panfrying without fat or oil, and oven cooking, on the color of steaks. The results showed that cooking methods significantly impacted the color of the steaks, with lightness (L*) and yellowness (b*) values increasing, while redness (a*) values decreased during cooking. The study concluded that cooking methods affected the color of steaks due to protein denaturation and losses of substances like minerals during cooking. Additionally, the study found that the highest mineral substance loss occurred with the microwave cooking method. Table 4 shows the color compounds of various types of red meat cooked using different cooking methods.

### 3.2. Chemical Properties

The cooking method and its conditions, such as temperature and cooking time, are reported as the most important factors affecting the chemical properties of fresh meat. One of the disadvantages of treating meat at high temperatures for long periods is the loss of vitamins, such as vitamins B1, B2, and C, as well as the loss of many minerals, such as zinc, iron, and calcium [83]. Moreover, Czerwonka et al. [84] reported that some food nutrients could be completely lost after cooking procedures. Nikmaram et al. [85] investigated the effect of the cooking method on the chemical composition, quality, and cooking loss of camel meat compared to veal. The results showed that moisture content decreases due to the high temperature, which causes protein denaturation and loss of minerals. Goran et al. [86] studied the effects of three different cooking methods (roasting, boiling, and microwave cooking) on the mineral content of beef and pork. The study found that cooking increased mineral concentrations in cooked samples compared to raw meat, with roasted samples showing the highest mineral concentration. When compared to pork, the amounts of trace elements in beef were greatly higher. The concentration of Na decreased in all samples of pork, indicating that Na has been lost with water, while Zn content in cooked beef samples showed significant differences from those of cooked pork. Oz et al. [66] investigated the effects of various cooking methods (deep fat frying, hot plate, boiling, microwave, pan-frying with oil, pan-frying without fat or oil, and oven cooking) on quality characteristics (water content, pH, and color values) and mineral composition of beef, as well as other properties of both raw and cooked samples. The study found that cooking methods had a significant impact on all the parameters studied, except for some mineral content (Fe, Mn, Ni, and Pb). The water content and all measured mineral levels decreased significantly. Furthermore, cooking resulted in the loss of less than 10% of the quantities of Fe, Pb, S, and Zn, in contrast to 13.6 to 21.1% for other minerals.

Moreover, the cooking process of meat has many advantages, the most important of which are enhanced meat palatability and improved digestion. Meat texture remains a critical aspect of meat-eating quality, which is significantly affected by temperature and cooking time through structural changes in meat tissue during different thermal processes [87]. The meat proteins denature and cause structural changes such as fiber shrinkage or aggregation, solubilization of collagen and connective tissues, and sarcoplasmic and myofibril gel formations [88].

### 3.3. Mechanical Properties

The mechanical properties of meat are important criteria that reflect the quality of meat. There are many devices for measuring mechanical properties, including the texture analyzer [89]. The texture of the meat can be defined as a set of properties that can be perceived by the mouth and eyes, and the texture profile analysis (TPA) is one of the most important mechanical tests for determining consumer acceptance of the food product. The TPA test involves pressing meat twice in a reciprocating movement that simulates jaw movement when chewing food [4].

There are many factors that affect the mechanical properties of meat, including temperature, time, cooking method, age of the animal, gender, and breed [28,90]. Many changes occur in the composition of the muscles when cooked at a high temperature. It was found that when meat is treated at a temperature of 50 °C, the protein fibers begin to deteriorate, while at a temperature of 60 °C, the muscle fibers begin to contract; at 70 °C, the contraction of muscle fibers increases more; at 80 °C, the breakdown and contraction of muscle fibers increase and the denaturation of collagen increases [6].

**Figure 6:**
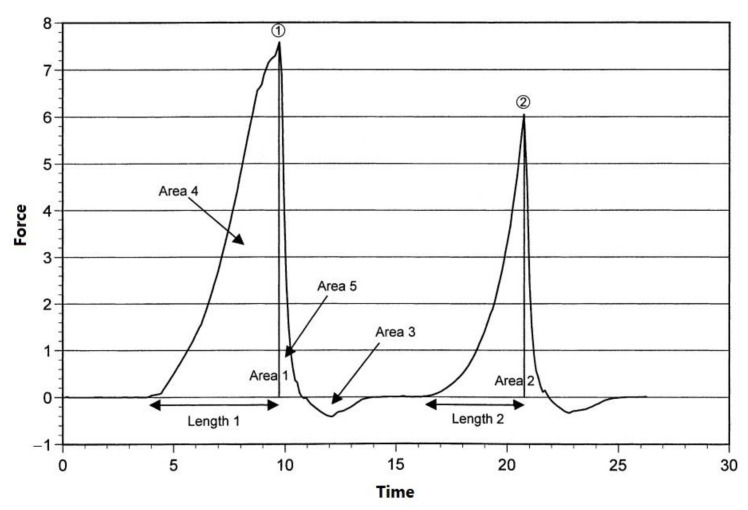
Typical texture profile analysis (TPA) of a *longissimus thoracis* rib steak [6].

Figure 6 illustrates the texture profile analysis curve of beef. The figure depicts the basic properties of TPA, namely hardness, cohesiveness, springiness, adhesiveness, and chewiness. Hardness (N) is defined as the peak of the maximum force ➀ during the first pressing cycle (first bite) and represents the force needed to maintain a specific defor-mation; cohesiveness is equal to Area 2/Area 1; springiness is defined as the distance that the sample recovers during the time between the end of the first bite and the beginning of the second bite ➁ and is equal to Length 2/Length 1; adhesiveness is the negative area under the baseline between the pressing cycles (bites) and is equal to Area 3; chewiness is defined as the product of hardness × cohesiveness × springiness [6].

There are many studies that have investigated the effect of cooking methods on the mechanical properties of meat, including that of James and Yang [72], who study the effect of cooking methods (oven, pressure cooking, vacuum cooking, and sous vide) on beef muscles. It was found that the highest value of shear force was in the oven cooking method due to the contraction of muscle fibers and the wrinkling of protein fibers. Nikmaram et al. [7] observed that when beef muscles are treated at a temperature of 70–80 °C, degradation and damage to some proteins occur, while at a temperature above 80 °C, damage occurs to most proteins. The denaturation of proteins is one of the most important factors affecting the tenderness of meat due to its effect on the hardness of the fibers. Table 5 presents the mechanical properties of some types of red meat cooked with different methods.

### 3.4. Thermal Properties

Information related to the thermal properties of food materials, including meat, is essential for researchers in the field of food processing, as this makes it possible to understand the temperature distribution within the food. Studying the thermal properties of meat depends on many factors, the most important of which are the temperature, the state of food and its chemical composition, the size and shape of the fibers, the moisture content, and the amount of fat [94,95]. The most important of these properties are the coefficient of thermal conductivity (*k*), the coefficient of thermal diffusion (α), and specific heat (*C_P_*).

The importance of studying thermal properties lies in the fact that they represent important engineering parameters in meat and food processing methods and play a pivotal role in the design of equipment [96]. It also determines the mechanism of thermal transfer within the product during processing [69].

The coefficient of thermal conductivity expresses the ability of a material to transfer or conduct heat through the thickness unit of the product if the temperature differs on the two edges of the unit thickness [97]. The coefficient of thermal conductivity is affected by several factors, the most important of which are temperature, components of the food material, and the location of the measuring probe, as it was observed that at high temperatures, there is an increase in the value of the coefficient of thermal conductivity [94]. The coefficient of thermal conductivity of meat can be measured in many ways, including the use of a thermal conductivity sensor, which is a circular cylinder with a high thermal conductivity coefficient in the form of a needle equipped with an insulated wire. Measurements are taken after the needle is inserted into the meat for measurement. This method has been used in many studies, including those by Hobani and Elanssari [94] and Pan and Singh [95].

The thermal diffusion coefficient of meat expresses its ability to distribute and diffuse heat to neighboring molecules or the ability of meat to absorb heat at a specified temperature during various processes [5]. Temperature, moisture content, and food material components are the most important factors affecting the thermal diffusion coefficient [97]. The coefficient of thermal diffusion of meat can be calculated using different devices or equations given the coefficient of thermal conductivity, specific heat, and density. Specific heat is defined as the thermal property that expresses the ability of the material to gain or lose heat, and the specific heat of meat can be determined with several methods, including the mixing method. In this method, the sample of meat is placed inside the calorimeter, and the specific heat is calculated with the equilibrium equation or by using the mass density of the product and the amount of total heat and temperature difference [98]. Furthermore, the value of specific heat can be calculated through the following relation:

(2) $$C_{p\;}=\frac{k}{\rho \times \alpha }$$

where *Cp* is specific heat, *k* is the thermal conductivity coefficient, (α) is the thermal diffusion coefficient, and (*ρ*) is density.

There are many studies that have investigated methods of cooking and their effect on the thermal properties of meat. In their study on some thermal properties of camel meat (Hashi) within the temperature range of 5–45 °C, Hobani and Elanssari [94] stated that the thermal conductivity coefficient of camel meat is (0.487) W/m. °C, and the thermal diffusion coefficient is 1.275 × 10^−7^ m^2^/s. Elansari and Hobani [96] also studied the effect of temperature and moisture content on the thermal conductivity coefficient of four types of meat (Hashi camel meat, Veal, Najdi, and Nuaimi) and noted that the coefficient of thermal conductivity has a linear relationship with the increase in temperature levels in the four types of meat, and the average coefficient of thermal conductivity ranged between 0.170–0.670 W/m. °C. Pan and Singh [95] studied the physical and thermal properties of minced beef during cooking in a study aiming to know the rate of change in the physical and thermal properties of meat inside a water bath at a temperature in the range of 40–70 °C for a time of up to 20 min. The results showed that the coefficient of thermal conductivity decreases with increasing temperature. Table 6 provides a summary of the thermal diffusion coefficient for various types of cooked red meat.

### 3.5. Sensory Properties

The quality standards for meat products are related to several sensory properties identified by food technologists and consumers, as consumers will accept specific food products but not others, and these properties include color, taste, smell, appearance, and texture, which can be perceived sensorially. Most researchers specializing in the sensory evaluation of food products prefer to use the sensory evaluation method, which relies on the subjective senses of the evaluators. Evaluators are highly trained to distinguish the flavor of the food product being evaluated and can sense small changes in the flavor of the product. Sensory evaluation is defined as a set of tests used to judge the quality properties of meat and is the result of a combination of physical and sensory properties such as color, tenderness, flavor, taste, and juiciness [104]. There are many factors affecting the sensory properties of meat, including the amount of connective tissue, the length of protein fibers, and the structure of different muscles, which has differences due to the age, sex, and diet of the animal [105].

#### 3.5.1. Tenderness

The tenderness of meat expresses the efficiency of breaking down meat by chewing in the mouth or the consumers’ perception that the structure of the meat is tender during chewing. It is considered one of the most important sensory properties that indicate the quality of meat to the consumer. The degree of tenderness of the meat can be determined by the assessors judging the ease with which the teeth penetrate the meat, the ease of separating the parts of the meat, and the amount of residue between the teeth. The tenderness of the meat is inversely proportional to the shear force, as the softer the meat, the lower the shear force [106]. There are several factors that determine the degree of tenderness of meat: the amount of connective tissue, the length of protein fibers, the amount of protein damaged after slaughtering the animal, the structure of different muscles, the method of cooking, and the size of the piece of meat [105].

During meat cooking, a set of changes occur in collagen, fiber, and tissue. There are several studies that are concerned with improving the tenderness of cooked meat, including that of Ha et al. [107] on the processes and cooking of meat to improve the structural properties and tenderness of meat. They stated that the tenderness of meat is one of the most important quality properties for consumers of each cut of meat and that the physical and chemical properties contribute to improving the texture and tenderness of meat. N’gatta et al. [75] also studied the effect of combining the method of sous vide cooking with added spices on the tenderness of beef, where the meat was cooked at 50, 60, and 80 °C for 1 and 4 h. Later, the shear force and cooking losses of the spiced and non-spiced meat samples were measured. The results showed that the shear force of the spiced samples decreased by 20% compared with the non-spiced samples. It has also been observed that the process of adding spices before cooking increases the tenderness of the meat.

Dominguez-Hernandez et al. [74] conducted a review of cooking meat at 50–65 °C for a long period of time, like sous vide cooking, where they observed that cooking at a low temperature over a long time increases the tenderness and improves the appearance of meat when compared to cooking at a high temperature. Vasanthi et al. [9] studied the effect of the cooking method (water bath and pressure cooking), cooking temperature (80–100 °C), and cooking time (30–60 min) on the sensory and structural properties of buffalo meat. The results showed that with increasing cooking temperature and time, the shear force decreased, and the tenderness of the meat increased. Moreover, there was no statistical difference in tenderness when cooking meat in a water bath or pressure cooker at 100 °C for 45 min.

#### 3.5.2. Flavor

Flavor is a mixture of sensations that include both the taste and smell of meat products. The clarity of meat flavor is related to many factors, the most important of which are the method and conditions of cooking, the age of the animal, and storage conditions. It is observed that the flavor of meat differs from one animal to another, and this difference is due to volatile substances associated with fatty tissue and streaky fat. The tongue is the organ responsible for distinguishing the taste of different foods, as there are specialized areas on the tongue to transmit the sense of taste, while smell is distinguished by the cytoplasm protrusions present in nose tissue [10].

Flavor is one of the most important factors that determine the degree of palatability of meat for the consumer, through which the consumer will also judge the quality of the meat. Different types of meat have a special flavor that distinguishes them from others, as the aqueous extract produced from muscle tissue contains free amino acids that play an essential role in giving the meat a distinctive flavor [48]. Chiavaro et al. [108] observed a direct relationship between the cooking method and the formation of volatile compounds, as these compounds affect smells and tastes experienced by the consumer.

#### 3.5.3. Juiciness

Juiciness is the amount of fluid in the mouth when chewing a food material, which depends on how moist the food is and the presence of juiciness fortifiers. The water and fat that exist in meat are considered the source of juiciness and can therefore affect the fibers’ capability to retain water. Fat stimulates the salivary glands in the secretion of saliva from the mouth and affects the sensation of juiciness [109].

Juiciness depends on the amount of moisture in the meat when chewing, as it plays an important role in conveying the taste to consumers. The juice contains many flavor ingredients that help assess the meat during chewing, as dry meat is less juicy and provides the feeling of roughness in the muscle fibers, giving the impression of hardness regardless of the degree of its juiciness [10]. Aaslyng et al. [67] noticed that when cooking pork chops in the oven at different temperatures (60–80 °C), there were significant differences in the juiciness of the cooked meat, and that juiciness decreased with increased temperature.

There are numerous studies that have been concerned with the sensory evaluation of cooked meat, including Mortensen et al.’s [48] research on the effect of long cooking times and low temperatures on the sensory properties of beef cooked with the sous vide method, where beef steaks were cooked at times of 3, 6, 9, and 12 h at temperatures of 56, 58, and 60 °C. The results showed that the sensory properties of cooked beef were acceptable when the meat was heated at 60 °C for 12 h. Das and Rajkumar [110] also studied the effect of different levels of fat on the sensory properties of goat meat cooked in a microwave, and the results showed that the cooking time decreased with the increase in fat content in the meat and a decrease in cooking losses was observed with a decrease in the fat content of goat meat. Ángel-Rendón et al. [22] investigated the effect of cooking methods (ohmic, sous vide, pan, and pressure cooker) on the structural, sensory, and physiochemical properties of pork, with the temperature at the center of the meat set at 70 °C for all cooking methods. The results showed that, at a 5% significance level, the tenderness, juiciness, and taste of meat cooked with pan and ohmic methods were similar, and consumers preferred meat cooked using those methods over other methods.

### 3.6. Microbial Properties

Meat is a perishable food material due to its high moisture content and the presence of many mineral elements, which makes it a suitable medium for the growth of microorganisms such as bacteria, fungi, and yeasts. The growth of microorganisms leads to undesirable changes in meat [111]. These are classified into changes in physical properties, such as discoloration, and chemical changes, such as changes in proteins and fats. There are many factors affecting the spoilage of meat, including temperature, moisture content, oxygen content, type of microorganisms, and the suitability of conditions for the growth of microbes.

There are many parameters that indicate microbial spoilage of meat, including the stickiness of meat due to the growth of *B. Bacillus* and *Lactobacillus* bacteria. Meat rot occurs due to the decomposition of proteins that leads to the formation of an undesirable flavor and smell due to the growth of *Pseudomonas*, *Lactobacillus* bacteria, and the fungi *Cladosporium* and *Aspergillus*. Changes in meat color occur due to the growth and reproduction of some microorganisms, which leads to the decomposition of pigments in the meat. Black spots are due to the fungus *Cladosporium*; white spots are because of *Sporotrichum;* a bluish-green color is due to the growth of the fungus *Penicillium* [112].

Adequate cooking of meat is essential to ensure that it is preserved, preventing spoilage, and that pathogens are eliminated. Meat cooking is one of the most important methods used to preserve meat [14,111]. Several researchers have highlighted the health risks of meat, as these risks are associated with the presence of toxin-producing bacteria at steady temperatures, which are selective aerobic bacteria [113]. Vaudagna et al. [51] stated that 50–60 °C is sufficient to kill vegetative microbes but not *B. clostridium*. Cayre et al. [114] also conducted mathematical modeling to investigate the growth rate of lactic acid bacteria in the emulsion of meat cooked in vacuum bags and stored at 0, 8, and 15 °C. They then studied the effect of storage temperature on the growth of lactic acid-producing bacteria under low-oxygen conditions at different storage temperatures. The Gompertz equation was used to predict the maximum growth rate of bacteria, and the results showed that the maximum growth rate of bacteria was at the temperature of 15 °C.

In another study, Zeleňáková et al. [115,116] assessed the microbial quality of meat products cooked during their shelf life. Microbial analysis of cooked pork and processed meats (sausages) was carried out during the storage period. The growth rate of Enterobacteriaceae and coliforms was analyzed, and the results showed that the highest growth rate of microorganisms in cooked meat was after 21 days, while in processed meat, it was 5 days within the temperature range of 2–6 °C. Yang et al. [117] also studied the effect of sequential sous vide cooking and cold storage conditions on the microbial properties of beef when it was cooked at 39 °C for one hour, then at 49 °C for one hour, then at 59 °C for 4 h, and then was stored for 28 days at −1.5 °C and 2 °C. The results showed that meat cooked using this method remained acceptable throughout the storage period. It was also noticed that during storage, the bacteria responsible for lactic acid did not grow. Xu et al. [118] also conducted a review on the uses of micro-preservatives in controlling the spoilage of beef and lamb and their impact on meat quality. They stated that these substances could be used to control the growth of undesirable microorganisms in fresh and cooked meat, as these substances can extend the shelf life of meat and maintain the sensory quality of meat.

## 4. Packaging and Shelf Life of Cooked Meat

The cooking process aims to preserve the quality of meat and its consumption in the best possible manner. The technique of cooking meat in vacuum bags is one of the modern technologies that have been recently spread [51,117]. The process of packaging meat started long ago to facilitate the process of transportation and storage and preserve the meat, preventing spoilage. Over time, the development of this technology has increased, and over the past two decades, different methods have been discovered to preserve meat, including the process of cooking meat in vacuum-sealed cooking pouches, known as Sous-vide Supreme. As mentioned earlier, this is considered one of the most important methods to save time and money and preserve the flavor and quality of meat for longer. Recently, there has been an increased demand for meat cooked in vacuumed plastic bags because it allows cooked meat to be easily handled without microbial contamination [51].

There are many factors affecting the process of packaging cooked meat, the most important of which is the type of packaging material and its tolerance to heat, as the packaging material consists of polymers that provide the basic structure to preserve the cooked product for as long as possible [119]. The packaging material for cooked meat must be resistant to high cooking temperatures and highly flexible to withstand sudden temperature drops below freezing [120]. It should also be airtight to prevent oxygen from entering and preserve the product for as long as possible, and it should be printable and highly heat sealable [121].Previous studies have shown that meat packaging before and after cooking extends the shelf life of meat up to 21 days due to a decrease in the oxygen content of vacuumed plastic bags and, thus, a decrease in the growth of aerobic microbes [51,116,117]. They also preserve the structural properties of the meat and its color hue, increase the tenderness of the meat, reduce the loss of water and mineral elements, and preserve the volatile flavors and odors [11,46,122].

The packaging and assessment of cooked meat and its shelf life have been investigated and evaluated by many studies, where the shelf life of cooked meat was strongly associated with several sensory and microbial properties. This includes the study of Cayre et al. [114] on the effect of storage temperature (0, 15, and 8 °C) and gas permeability on the growth of lactic acid bacteria and *Brochothrix thermosphacta* bacteria in the emulsion of meat cooked in vacuumed bags. Several predicted variables from the Gompertz equation and the analysis of the effect of packaging permeability and temperature on the growth rate of bacteria were used, and the results showed a significant effect of the factors at the level of (*p* < 0.001). Horita et al. [123] conducted a review of the combination of packaging cooked meat and using non-thermal treatments to remove microbial contamination of ready-to-eat cooked meat. In their study, they stated that cooked meat is damaged by *Listeria monocytogenes* microorganisms, which appear after the cooking process and grow rapidly, leading to meat deterioration. Therefore, some non-thermal techniques are used, such as ultraviolet light and pulsed electric field, to eliminate decontamination and microorganisms after packaging and reduce the activity of pathogenic microorganisms present on the surface of cooked meat.

## 5. Mathematical Modeling

Meat cooking can be mathematically modeled by considering the heat transfer and mass transfer processes that occur during cooking. Mathematical modeling of the meat cooking process is essential for understanding heat and mass transfer phenomena, designing devices, maintaining the quality of consumed meat, reducing production costs, improving meat quality, and extending the shelf life of meat. Mathematical models consist of a set of mathematical equations or relationships that describe the meat cooking process. These models are used to simulate or predict the behavior of the meat cooking under different conditions and can be used to optimize or control the cooking process parameters such as the cooking time, internal temperature, and final texture and flavor of the cooked meat. However, it is important to validate the results of the simulations with experimental data to ensure accuracy and reliability [124].

Typically, when cooking meat in an oven, heat is transferred from the surrounding environment to the surface of the meat through convection and radiation. This causes the surface of the meat to become hot, and heat is then conducted towards the center of the meat. Internal moisture is transported toward the meat surface by diffusion and convection mechanisms and lost to the air by evaporation [125].

The governing equations for heat and mass transfer during meat cooking can be completed due to the involvement of various physical factors, including heat and energy transport, mass transport, fluid flow dynamics, and mechanical changes such as shrinkage and swelling [13]. The heat transfer (conduction and convection) within the meat is governed using the energy conservation equation [126]:

(3) $$\rho c_{p}\left(\frac{\partial T}{\partial t}\right)+\nabla .\left(-k\nabla T\right)+\rho_{w}c_{p,w}u_{w}\nabla .T=0$$

where ρ, $c_{p}$, and k are the density (kg/m^3^), specific heat (J/kg·K), and thermal conductivity (W/m·K) of the meat, respectively, $\rho_{w}$, $c_{p,w}$, and $u_{w}$ are the density (kg/m^3^), specific heat (J/kg·K), and thermal conductivity (W/m·K) of the liquid transported within the meat, respectively, and T is the temperature (C°), and *t* is time cooking (sec).

The governing equation of mass transfer (by diffusion and convection) within the meat is based on the mass conservation equation [126]:

(4) $$\frac{\partial C}{\partial t}+\nabla .\left(-D_{w}\nabla C+u_{w}C\right)=0$$

where *C* is moisture concentration (mol/m^3^), and *D_W_* is the moisture diffusion coefficient (m^2^/s) in the meat.

Darcy’s law can be used to describe the velocity of the fluid in the pores within the meat from the conservation of momentum:

(5) $$u_{w}=\frac{-x}{\mu_{w}}\nabla p$$

where *x* is the permeability of the meat (m^2^), $\mu_{w}$ is the dynamic viscosity of the fluid (Pa·s), and ∇p is the pressure gradient vector (Pa/m).

Overall, the most accurate models for predicting cooking times will take into account the specific cooking method, the cut and thickness of the meat, and any other relevant factors that may affect the mass and heat transfer and cooking process. There have been many attempts to design mathematical models for cooking meat where several influencing factors have been ignored, including stickiness, contraction, and elasticity, and many equations have arisen based on the principle of conservation of mass, energy, and balance of forces [124].

Many studies have conducted mathematical modeling of meat during cooking, including the study of Ahmad et al. [127], where a mathematical model was developed for the process of cooking minced meat using the C++ program, and the meat was cooked in a cylinder with a diameter of 6.6 cm, a length of 18 cm, and a weight of 650 g. In this study, the temperature of the cylinder rose during natural and forced convection, as the mixing of cold air and boiling temperatures was predicted, and the mathematical model was solved using the Rung–Kuta fourth-order equation. The results obtained from the model were compared with the laboratory values. Many of the assumptions necessary for mathematical modeling were relied upon, including the use of thermal diffusion and moisture as constants, and the fat transfer was neglected and based on the above assumptions. The mathematical model describes the heat and mass transfer during the cooking of minced meat in a cylinder well. In another study, a 3D computational model was developed using COMSOL Multiphysics 5.2a to simulate heat and mass transfer, as well as deformation, during steak cooking on a double-sided pan with different cooking times and meat thicknesses. The results showed that the model accurately predicted the central temperature and cooking time of the steak with root mean squared errors of 2.16 °C for very rare, 3.56 °C for rare, and 4.57 °C for well-done cooking, and 1.48%, 2.08%, and 2.40% water loss, respectively. The authors concluded that the developed model is a useful tool for improving cooking processes in the food industry, helping to improve the consistency and quality of cooked meats [128],

Experimental mathematical relationships have also been developed on water retention and the storage coefficient. In another study, Nelson et al. [124] performed mathematical modeling of cooked beef where a two-dimensional mathematical model of steaks was utilized cooking using the Flory–Reihner theory, and this method depends on the presence of an elastic medium saturated with liquids. It was observed that with the rise in cooking temperature, deformation of the meat protein occurs, which leads to water and mineral elements leaving, causing shrinkage in the piece of meat during cooking; it has been observed that the piece of meat shrinks up to 30% during cooking. Table 7 shows some mathematical models used in various studies about meat cooking.

## 6. Conclusions

This review can be summarized as follows:

1. When red meat is processed at high temperatures, several changes occur in the engineering properties and structural quality. There are many factors affecting these properties, the most important of which are the method of cooking and cooking conditions like heating rate, cooking time, and temperature;
2. Determining the engineering properties of meat before and after cooking is essential to establish the extent of consumer acceptance of meat and identify the composition of meat before and after the cooking process. The engineering properties of food materials play an important role in understanding the process of heat and mass transfer to and from a food material. The quality standards for meat products are also linked to many sensory and microbial properties that have been determined by food technologists and consumers so that the consumer will accept a specific food product but not another. These properties include taste, smell, texture, and appearance, which can be perceived sensorially;
3. The importance of mathematical modeling of the meat cooking process lies in the rapid development of various cooking techniques, creating the need to reduce the costs of laboratory experiments and understand the phenomena of heat and mass transfer. This allows for the possibility of maintaining the quality of meat, reducing production costs, and improving the quality of meat flavor;
4. There are many methods that were not addressed in this scientific review. However, they have great research potential since they can be combined using hurdle technology to eliminate the highest ratio of microorganisms, obtain high quality, and reduce the rate of energy consumption.

## Figures and Tables

**Table 1:** Synopsis of previous studies investigating different types of red meat and cooking methods.

Type of Meat	Cooking Method	Cooking Conditions	Highlights	Reference
Camel meat *(Latissimus dorsi)*	Sous vide and electrical oven cocking	70, 80, 90, and 100 °C and 30, 60, 90, 120, 150, and 180 min	The study showed an increase in pH, cooking loss, and yellowing color compounds and a decrease in density, water activity, lightness, and redness. The sensory properties showed an increase in tenderness and flavor but a decrease in juiciness. The study concluded that the best cooking method for the meat is sous vide at a temperature of 100 °C for 180 min.	[19]
Pork loins *(M. longissimus)*	Sous vide	60 °C and 65 °C for 2, 3, and 4 h, and 70 °C and 75 °C for 1, 1.5, and 2 h	The cooking loss increased with increasing temperature and cooking time but had no effect on pH and water activity values. Pork cooked at 60 °C or 65 °C for 4 h showed more tenderness and flavor compared to other cooking temperatures.	[20]
Beef strip loins *(longissimuslumborum)*	Grilling and oven roasting	150 °C and 230 °C	Grilled dry-aged beef had a stronger roasted flavor than that cooked in an oven. Moreover, grilling dry-aged beef at 150 °C resulted in a higher intensity of cheesy flavor, whereas at 230°C resulted in a greater intensity of roasted flavor compared to wet-aged beef. Hence, grilling can be considered a promising cooking method for improving the flavor of dry-aged beef.	[21]
Pork (short shank)	Sous vide and ohmic cooking	70 °C	The results showed that the tenderness of the meat increased in all ways and thus increased the acceptance of sensory assessors. There were no significant differences between the different methods of cooking losses and water holding capacity.	[22]
Beef steaks	Electrical and Gas Oven	Up to 110 °C	The study found that protein and water contents and physical properties of electric and gas oven-cooked smoked meats were similar. Electric oven-cooked smoked meats had higher fat content but lower TBA and peroxide values compared to gas oven-cooked smoked meats.	[23]
Foal steaks	Frying	150–190 °C	The results showed that the cooking losses and the shear force in the microwave cocking were higher, followed by frying, while the color compounds were higher in fried horsemeat using olive oil.	[24]
Ground beef	Ohmic cooking	20, 30, and 40 V/cm	The results showed that ohmic cooking was faster than traditional cooking at a significance level (*p* < 0.05). Moreover, ohmic cocking was more stable and coherent compared to conventional cocking.	[25]
Beef steaks	Atmospheric pressure, sous-vide, and cook-vide	60, 70, and 80 °C 15, 30, 45, and 60 min	Sous-vide and cook-vide produced beef that retained more moisture and tenderness compared to atmospheric pressure cooking. In addition, sous-vide cooked beef exhibited superior color retention due to the oxygen-depleted cooking environment compared to atmospheric pressure cook-vide cooking.	[4]

**Table 2:** pH level of some types of red meat cooked using various techniques.

Type Meat	Cooking Method	Cooking Conditions	pH Level	References
Beef (*M. Longissimus dorsi*)	Frying	200 °C, 6 min	5.66	[66]
	Oven	200 °C, 9 min	5.62	
	Microwave	4.5 min	5.63	
Carabeef *(Semimembranosus)*	Water bath	100 °C, 60 min	6.48	[9]
Camel *(Latissimus dorsi)*	Sous vide	70–100 °C, 30–180 min	6.04–6.57	[19]
	Oven		5.90–6.44	
Beef- meatball	Ohmic	140 V	5.16	[64]

**Table 3:** Cooking loss for some types of red meat cooked in different cooking methods.

Type Meat	Cooking Method	Cooking Conditions	Cooking Loss, %	References
Goat *(Semimembranosus)*	Sous vide	50 °C–90 °C	5.91–41.25	[71]
Bovine *(M. Semitendinosus)*	Electric Oven	200 °C, 15 min	31	[72]
	Sous vide	60 °C, 60 min	19	
Beef-meatball	Ohmic cooking	75 °C	15.75	[73]
Beef *(Longissimus dorsi*)	Electric Oven	110 °C, 15 min	13.6	[23]
	Gas oven		11.79	
Foal *(Longissimus dorsi)*	Microwave	1000 W, 1.5 min	32.49	[74]
	Frying	180 °C, 4 min	23.73	
	Electric oven	200 °C, 12 min	26.71	
Camel *(Latissimus dorsi)*	Sous vide	70–100 °C, 30–180 min	31.87–48.56	[19]
	Oven		1.52–46.1	
Bovine *(Semitendinosus)*	Sous vide	80 °C, 4 h	45	[75]
Pork loins *(M. longissimus)*	Sous vide	70 °C, 2 h	30.58	[20]

**Table 4:** Color compounds for some types of red meat cooked using different techniques.

Type Meat	Cooking Method	Cooking Conditions	L*	a*	b*	References
Beef (*M. Longissimus dorsi*)	Frying	200 °C, 6 min	41.32	13.48	6.60	[66]
	Oven	200 °C, 9 min	41.42	14.21	6.14	
	Microwave	4.5 min	41.74	14.17	6.23	
Camel *(Latissimus dorsi)*	Sous vide	70 °C–100 °C, 30–180 min	55.4–30.9	14.2–7.1	15.6–3.29	[19]
	Oven		55.4–31.8	17.8–4.12	15.6–3.3	
Lamb *(Longissimus dorsi)*	Microwave	70 °C	48.44	12.71	11.45	[81]
Beef *(Transversus thoracis)*	Sous vide	60 °C–70 °C, 12–36 h	51.5–45	11.5–11.2	16.1–14.1	[82]
Pork loins (*M. longissimus)*	Sous vide	70 °C, 2 h	70.81	7.86	13.46	[20]

**Table 5:** Mechanical properties of some types of red meat cooked in different ways.

Type Meat	Cooking Method	Cooking Conditions	Shear Force (N)	Hardness (N)	References
Veal *(Longissimus dorsi)*	Microwave	100 °C	37	-	[7]
Camel *(Latissimus dorsi)*	Sous vide	70 °C–100 °C, 30–180 min	60.9–14.4	20.5–4.6	[91]
	Oven		59.9–28.3	13.9–0.3	
Beef *(Deep pectoral)*	Sous vide	80 °C, 60 min	60	-	[4]
Bovine *(M. Semitendinosus)*	Oven	200 °C, 15 min	100	-	[72]
	Sous vide	60 °C, 60 min	75	-	
Beef (*Transversus thoracis*)	Sous vide	60 °C–70 °C, 12–36 h	19.3–18.9	21.9–17.9	[82]
Beef *(biceps femoris)*	Sous vide	65 °C, 8 h, 12 h	47.5–43.6	29.7–25.8	[92]
Beef *(longissimus thoracis)*	Sous vide	65 °C, 2.5 h	20.4	-	[93]

**Table 6:** Thermal diffusion and thermal conductivity for different types of processed red meat.

Type Meat	Cooking Method	Thermal Diffusion Coefficient (m^2^/s)	Thermal Conductivity (W/m.K)	References
Beef *(longissimus)*	Grill	0.23 × 10^−7^–0.25 × 10^−7^	0.55–0.57	[69]
Beef *(strip loins)*	Grill	0.16 × 10^−7^–0.18 × 10^−7^	0.48–0.47	[99]
Camel *(Latissimus dorsi)*	Sous vide	1.46 × 10^−7^–1.16 × 10^−7^	0.51–0.37	[91]
	Oven	1.4 × 10^−7^–1.17 × 10^−7^	0.51–0.36	
Lean pork *(leg muscle)*	Frying	0.24 × 10^−7^–0.25 × 10^−7^	0.79–0.35	[33]
Beef-burger	Oven	-	1.22–1.82	[100]
Beef- meatball	Frying	0.287 × 10^−7^	1.33	[101]
Ground beef	Water bath	-	0.35–5.41	[95]
Mortadella	Oven	2.4 × 10^−7^	-	[102]
Sausage	Frying	3.85 × 10^−7^–1.31 × 10^−7^	-	[103]

**Table 7:** Mathematical models used to solve some red meat cooking in recent years.

Meat Cooking Study	Mathematical Model	Results	Reference
Optimization of beef heat treatment using CFD simulation: Modeling of protein denaturation degree	Computational fluid dynamics (CFD) simulation	The CFD model accurately predicted the degree of protein denaturation and can be used to optimize the heat treatment process of beef, resulting in a more consistent and high-quality product.	[129]
Color changes in beef meat during pan cooking: kinetics, modeling and application to predict turn over time	The finite element method through COMSOL Multiphysics	The developed mathematical model effectively predicted the time required to achieve a desired color change in beef during pan cooking.	[130]
A Mathematical model for meat cooking	The finite difference equations using Flory-Rehner Theory with C++	A mathematical model for meat oven roasting was developed, accounting for the effect of shrinkage on the cooking process. The results of the numerical simulations demonstrated a good agreement with the experimental data.	[131]
Model for electrical conductivity of muscle meat during Ohmic heating	Empirical equations	A mathematical model was developed and validated to accurately simulate the changes in the electrical conductivity of muscle meat during ohmic heating.	[132]
Mathematical modeling of ground beef in a cooking cylinder	The finite difference equations through the fourth-order Runge-Kutta method with C++	A mathematical model was developed to predict the temperature history of meat cylinders during different cooking conditions. Temperature predictions were in agreement withexperimental values.	[127]
The ohmic heating of meat ball: Modeling and quality determination	Sukprasert’s model and an adjusted finite difference model	The Sukprasert and the adjusted finite difference models used in this study were the most precise to accurately predict the changes in temperature of pork meatballs during ohmic heating.	[133]
Prediction of cooking times and weight losses during meat roasting	The finite element method using COMSOL Multiphysics and MATLAB	The developed model was validated using experimental data, and it was found to accurately predicts cooking times and weight losses for beef cooked in an oven.	[134]
Solid food pasteurization by ohmic heating: Influence of process parameters	The finite element method using COMSOL Multiphysics	A previously developed model was successfully used to investigate the influence of meat pasteurization process parameters on temperature distribution, resulting in a uniformly pasteurized product	[135]

## Data Availability

No new data were created or analyzed in this study. Data sharing is not applicable to this article.
